# Total Number Is Important: Using the Disector Method in Design-Based Stereology to Understand the Structure of the Rodent Brain

**DOI:** 10.3389/fnana.2018.00016

**Published:** 2018-03-05

**Authors:** Ruth M. A. Napper

**Affiliations:** Brain Health Research Centre, Department of Anatomy, School of Biomedical Sciences, University of Otago, Dunedin, New Zealand

**Keywords:** stereology, serial block-face scanning electron microscopy, total number, rodent brain, the disector method

## Abstract

The advantages of using design-based stereology in the collection of quantitative data, have been highlighted, in numerous publications, since the description of the disector method by Sterio (1984). This review article discusses the importance of total number derived with the disector method, as a key variable that must continue to be used to understand the rodent brain and that such data can be used to develop quantitative networks of the brain. The review article will highlight the huge impact total number has had on our understanding of the rodent brain and it will suggest that neuroscientists need to be aware of the increasing number of studies where density, not total number, is the quantitative measure used. It will emphasize that density can result in data that is misleading, most often in an unknown direction, and that we run the risk of this type of data being accepted into the collective neuroscience knowledge database. It will also suggest that design-based stereology using the disector method, can be used alongside recent developments in electron microscopy, such as serial block-face scanning electron microscopy (SEM), to obtain total number data very efficiently at the ultrastructural level. Throughout the article total number is discussed as a key parameter in understanding the micro-networks of the rodent brain as they can be represented as both anatomical and quantitative networks.

## Introduction

The first detailed drawings of the cells of the brain, by “Ramón y Cajal” indicated that the brain is a complex arrangement of many different types of cells (see Swanson and Lichtman, 2016). Modern imaging methods have shown that the complexity of this network spans from the nanoscale to axonal pathways (Bohland et al., 2009; Finlay, 2016). This network is formed by the connections between a series of nodes, at cellular and subcellular levels, each representative of a specific brain region and fundamental to functional interactions in the brain (Barbas, 2015; Li et al., 2016). Within each node there is a total number of cells and synapses and these numbers are crucial in understanding how a brain region interacts functionally with other regions. All anatomically connected brain regions are quantitative networks and models of normal and pathological brain function benefit from using estimates of total number (for early descriptions of this idea see Mulders et al., 1997). The total number of neurons and/or synapses in each region, each node, in a quantitative network must be obtained without size or shape bias and within identifiable anatomical boundaries (Witter, 2010). The unbiased stereological estimator, the disector, combined with design-based stereological sampling can be used to obtain an estimate of total number within a defined volume of tissue (West et al., 1991; Oorschot, 1996).

Rodents, particularly rats and mice, are key animal models used to investigate many pathologies of the human brain (Nestler and Hyman, 2010) and thus it is essential to have quantitative models of the rodent brain networks to correlate with function. This review article will focus on the idea that total number, either of cells or synapses, obtained with the disector method, is crucial in enabling the rodent brain to be understood as a quantitative network that allows both function and dysfunction to be interpreted at a cellular basis. Finally, it will suggest that the disector method is poised along-side developments in electron microscopy to allow major advances in our understanding of the quantitative structure of the rodent brain at the subcellular level. Throughout the article total number is discussed as a key parameter in understanding the micro-networks of the rodent brain as they can be represented as both anatomical and quantitative networks.

## The Disector Method—A Revolution in the Estimation of Total Number

The brain is comprised of a number of component networks of individual cells and synapses that can be considered particles with a certain shape and size. These particles can be counted as long as any profile of the particle can be reliably identified in random sections through the particle. It has been well established that within the brain both cells and synapses rarely conform to simple geometric shapes and that the volume of particles may change with experimental treatment as noted in a number of studies (Kitahara et al., 2016). If number is estimated from the presence of particles in a single tissue section it is biased towards particles of larger volume and although a range of correction factors can be applied to single section estimates this is far from ideal (Calverley and Jones, 1987; Calverley et al., 1988; Park and Ahmad, 2014). The existence of a particle size bias should have been abolished with the publication of a seminal article describing the disector method in 1984 by Sterio. This method was a major breakthrough in quantitation. It requires that all profiles from a particle can be identified in a section, that each particle has a single “top” or “bottom”, depending on the direction from which slices through the particle are viewed and that section thickness is known, preferably measured. The original disector method (Sterio, 1984), known as the physical disector used two adjacent sections a known distance apart, that was not greater than around one third of the height of the smallest particle to be counted. Particle profiles visible in one section, the reference section, are counted if they are not present in the parallel “lookup” section, meaning the profile counted must have been either the top or bottom of the particle. This method and how to apply it over a range of cell and synapse types are described in a number of excellent books, key stereological reviews and specific research publications (Geinisman et al., 1996; Howard and Reed, 2005; West, 2012, 2013a; Mouton, 2014). The disector method was rapidly adopted by neuroscientists estimating synapse number as serial physical sections are commonly used in transmission electron microscopy and it was well established that synapse size and shape varies with section orientation (Geinisman et al., 1992, 1996). It continues to be a key tool for estimating total synapse number and synapse number per neuron (da Costa et al., 2009; Jasinska et al., 2016).

The use of two parallel sections to form the disector was ideally suited to transmission electron microscopic studies of subcellular elements but was time consuming at a light microscope level. The optical disector method removed this constraint by using one relatively thick physical section and counting within it, through a series of optical sections (Gundersen, 1986). Essentially particle number is counted within an unbiased sampling frame as one focuses through a series of optical sections for a known distance that forms the z-dimension of the disector volume. In three dimensions the unbiased sampling frame is an unbiased sampling volume with either the upper or lower optical section forming an exclusion plane (Gundersen, 1986; Gundersen et al., 1988a; West et al., 1991; West, 2013a; Mouton, 2014). This method requires accurate measurement of the z-direction within the section and use of a high-quality objective lens to minimize the thickness of the focal plane. Optical section thickness can be minimized in the confocal microscope making it ideally suited for the optical disector method (Peterson, 1999; Kubínová and Janáček, 2015). Detailed explanations of the optical disector method can be found in key stereological articles (West, 2013a; Mouton, 2014) and examples of its use in specific brain regions are numerous (West et al., 1991; Oorschot, 1996; Bonthius et al., 2004; Ash et al., 2014).

The disector method provides an unbiased estimate of total number within the disector volume sampled within the structure of interest. This is a measure of density (*N*_v_) within the known disector volume but if the total volume of the structure is not known this can be misleading (Oorschot, 1994; Coggeshall and Lekan, 1996; Dumitriu et al., 2012; Ash et al., 2014). The volume of the brain region within which density is estimated may change due to tissue processing or many other factors and this change may be differential across experimental groups and to an unknown extent. The relationship between density and total number is unknown, thus making density a potentially misleading quantitative measure. This is avoided by determining the volume of the region of interest, the reference volume (Vref), within which the density is determined. These parameters can then be used to estimate the total number (*N*) where *N* = *N*_v_ × Vref. These methods are described in detail in many excellent sources (Gundersen et al., 1988b; West, 2013a; Mouton, 2014).

The removal of potential bias but also efficiency have been important concerns as stereological methods have continued to be developed (Gundersen and Jensen, 1987; Gundersen et al., 1999; West, 1999, 2012; Hosseini-Sharifabad and Nyengaard, 2007). The unbiased sampling design of stereology, based on application of a set of uniform random points, enabled concurrent estimation of Vref during a disector estimation of density. This had the advantage that the estimate of total number (*N*) is not affected by any change in tissue volume as all the measurements are relative, are fractions (Gundersen et al., 1988a; West, 1993, 2002). The disector method, following a random systematic sampling design, estimates particle number (*N*) from the number of particles counted (∑Q^−^) in a known volume of tissue that is a known fraction (f) of the volume of region of interest (the Vref). The total number (*N*) of particles within the Vref is determined from the number of particles counted (∑Q^−^) and the inverse of the fraction (f), *N* = ∑Q^−^ × (1/f). The original method used the physical disector but with use of the optical disector the method became known as the optical fractionator (West et al., 1991; West, 2002; Hosseini-Sharifabad and Nyengaard, 2007) and is very widely used. Despite the unbiased nature of the disector method and the major advantages of using an estimate of total number, the methodology still contains potential sources of systematic bias. With increased use of the optical disector over the last decades the type of sections within which a disector density estimate is made has changed and become more diverse. Tissue deformation during sectioning and the potential loss of particles from the section surface are long standing issues, addressed by assigning an upper and lower guard to each section where particles were not counted (Gundersen, 1986; West et al., 1991). However, there is evidence of uneven section shrinkage in frozen sections, an increasingly common choice for optical disector studies (Bonthius et al., 2004; Carlo and Stevens, 2011; Puigdellívol-Sánchez et al., 2015) and of variable density along the z-axis in sections from a range of sectioning methods (Hatton and von Bartheld, 1999; Dorph-Petersen et al., 2001; Gardella et al., 2003; von Bartheld, 2012; West, 2013b). These potential sources of systematic bias are important and solutions such as using smaller counting frames that vary in position in the Z-axis, have been suggested as ways of achieving higher precision (Puigdellívol-Sánchez et al., 2015). Investigators should publish information on the tissue processing, embedding and cutting protocols used, alongside details of stereological sampling parameters so that results can be compared across research studies. Neuroscientists would benefit if journals insisted these parameters be included in publications as it would allow data to be more easily compared between studies. Other sources of potential bias, such as differences between experimenters in object or boundary identification can be minimized by including specific criteria used and photographic evidence (West et al., 1991; Gondré-Lewis et al., 2016). Immuno-labeling to phenotype particles of interest, can help identification but antibody penetration and labeling success in the z-axis direction must be assessed and reported (Ash et al., 2014). Despite the potential of errors within individual estimates of total number, total number remains superior to density estimates and can be combined with our knowledge of brain networks to develop quantitative networks at both cellular and synaptic levels of anatomically connected regions within the rodent brain. This type of network will enhance our understanding of the rodent and thus the human brain (Gulley and Juraska, 2013 and see DeFelipe, 2015; DeFelipe et al., 2016 for more detailed discussion).

## Total Number—Not Volume or Density—the Past

It has been over three decades since the original description of the disector method and the demonstration that volume and density on their own were of limited value in understanding the number of structures within a brain region. One of the most influential studies that emphasized the importance of using total number within a defined volume, not density, was an early study undertaken by stereologists on human brain tissue. A 40% decrease in total neuron number was found in the mediodorsal thalamic nucleus from schizophrenic patients compared to controls despite numerous previous studies reporting no change in density (Pakkenberg and Gundersen, 1989). The potential effect of a volume change on density had been ignored and misleading data resulted. Total number via a disector estimation, not an unbiased density estimate alone, has been the key major advance in understating the rodent brain in health and disease. An understanding of the value of total number estimates within a defined brain region has provided understanding of a key neuroscience dogma; “that cells are lost from the brain as it ages”, which contained the implication that this occurred throughout the brain. This view had arisen because density estimates, made within unknown tissue volumes, had resulted in misleading data. Although there was no significant decrease in the total number of neocortical neurons in the aging human brain (Pakkenberg and Gundersen, 1997) neuronal deficits have been found in some specific cortical regions and layers and for some neuronal phenotypes during aging (Shi et al., 2006; Stranahan et al., 2012). It has also been found that there is an age-related decrease in the total number of granule and Purkinje cells in the anterior, but not posterior lobe, of the cerebellum (Andersen et al., 2003). This study also found the volume of the Purkinje cell perikarya decreased with aging, emphasizing the importance of the disector method in determining density without bias from cell size (Andersen et al., 2003). The lack of neuronal loss in the hippocampus and key output regions stimulated alternative areas of investigation on cognitive decline in aging (Merrill et al., 2001). Stereological quantitation combined with immuno-labeling found a specific loss of GAD67- and SOM-positive neurons in the hilar region of memory impaired rats but no decline in total number of NeuN labeled cells. This finding was instrumental in associating a loss of protein expression in the labeled cells with dysfunction suggesting therapeutic intervention may be possible (Spiegel et al., 2013). Concurrently the total number of glial cells in specific brain nuclei increases during aging stimulating further research on the role of glial cells as causative rather than reflective of brain changes (Rubinow and Juraska, 2009). It is clear that aging can alter the total number of neurons within specific brain regions but the important point is that use of the disector method, ensures that the density estimate is not biased by age-related particle size changes, plus the conversion of sample density to total number, accommodating unknown regional volume change, has enabled the generation of reliable findings (Pakkenberg and Gundersen, 1989, 1997). Total number estimates within design-based stereological studies have contributed in past decades over the breadth of neuroscience to advance our understanding of Parkinson’s and Huntington’s disease (Arcuri et al., 2016), ischemeic brain injury (Avendaño et al., 1995; Mestriner et al., 2013), epilepsy (Foresti et al., 2009; Ye et al., 2013), schizophrenia (Kaalund et al., 2013), depression (Allard et al., 2004) and traumatic brain injury (Bregy et al., 2012; Cope et al., 2016) to name a few. Understanding injury in the developing brain has also been advanced by the use of total number with studies of hypoxic/ischemeic brain damage (Cameron et al., 2015), fetal alcohol spectrum disorder (Napper and West, 1995; Klintsova et al., 1997), maternal stress (Oreland et al., 2010) and a range of other prenatal insults (Smith et al., 2008; Sadowski et al., 2014). The endpoint of the majority of this research is to understand brain function. Thus, investigation of the total number of synapses in a brain region or on a specific type of neuron is as important as total cell number in understanding how neuronal populations interact within a network (DeFelipe, 2015). As mentioned previously, estimation of the total number of synapses with the disector method was rapidly adopted (Geinisman et al., 1992, 1996). Numerous studies have used disector estimates of total synapse number to investigate changes resulting from a number of phenomena including protein undernutrition (Lukoyanov and Andrade, 2000), hypothyroidism (Madeira and Paula-Barbosa, 1993), aging (Poe et al., 2001), epilepsy (Thind et al., 2010; Yamawaki et al., 2015), diabetes (Zhao et al., 2016) and in some cases the reversibility of synapse change (Lukoyanov and Andrade, 2000; Yamawaki et al., 2015). Studies have also indicated that an increase in the number of synapses occurs with a change in behavior (Klintsova et al., 1997; Hajszan et al., 2009; Dalzell et al., 2011; Jasinska et al., 2016). An important aspect of a disector estimate of total synapse number is that values obtained from control animals within any study can be used, due to the unbiased nature of the estimator, by other investigators to model and understand connectivity in the rodent brain (da Costa et al., 2009; Ciccarelli et al., 2012). Other provisos do affect how comparable such data sets are but when provisos are noted, total number becomes valuable to the global neuroscience community in a way density cannot be, even if obtained with the disector method.

## Total Number—the Present

As indicated above the ways in which design-based stereological estimates of total number have contributed to our understanding of the rodent brain is immense but this may be under threat due to considerable pressure to generate data as efficiently as possible. Efficiency has always been a key focus of the expert stereologists who have driven the ongoing development of stereological methods (Nava et al., 2014) with research publications also stressing the efficiency of design-based stereology (West et al., 1991; Johnson, 2001; Zhu et al., 2015; Kelly and Hawken, 2017). However, a more recent method to obtain total number estimates, the “isotropic fractionator” is gaining popularity as studies show that under certain conditions it generates data comparable to stereological based total number estimates but in less time (Herculano-Houzel and Lent, 2005; Herculano-Houzel et al., 2015). However, this method has major limitations. One mentioned by Herculano-Houzel (2017), is that the process of homogenization destroys all information about the structural arrangement of cells within the tissue which is extremely important in understanding how function emanates from a biological region. Another serious limitation is that the brain region of interest must be accurately dissected out, prior to homogenization precluding obtaining information from small brain regions, layers and at an ultrastructural level (Fu et al., 2013). This may also result in considerable inter-investigator variability. If we consider that a key use of total number, obtained using design-based stereology, is to understand the relationship between cells, the quantitative network of the brain, this method will not deliver.

Immuno-labeling methods have been an essential tool in design-based stereological studies and have provided total number data on specific cell types (Mokin and Keifer, 2006; Prasad and Richfield, 2010). The continual development of imaging technologies and the minimal thickness of confocal imaging planes combined with immuno-labeling allows precise identification of molecular species and quantitation with a precision previously only possible in the electron microscope (Peterson, 1999). The use of transgenic mice is also an opportunity where design-based stereological determinations of total number could make a major contribution to understanding the phenotype of these animals and changes in brain structure and function (Berlanga et al., 2011; Manaye et al., 2013; Manaye and Mouton, 2014). However, despite many publications highlighting the “reference trap”, the volume within which the disector estimate was made is not always determined and data is presented as “densities” which may be misleading, as discussed above (Siucinska et al., 2014; Woeffler-Maucler et al., 2014). It is also important to note that many of these studies do not report the use of systematic random sampling, a requirement of design-based stereology to ensure the estimate is representative of the whole tissue region, not just the sample area, despite this capability existing in most confocal imaging platforms. Neuroscience has already seen that biased data can direct science down false avenues and waste considerable research resources (Pakkenberg and Gundersen, 1989; Mura et al., 2004). It is also important to emphasize that the use of unbiased stereology to estimate total number is not as time consuming as naïve investigators may think (Gundersen and Osterby, 1981; Gundersen and Jensen, 1987; West et al., 1991; Brasnjevic et al., 2013; Wang et al., 2014).

## Total Number—the Future

Faced with trying to understand the rodent brain, many neuroscientists consider that the total number of neurons/synapses is important and that reliable estimates of such values are an essential requirement of quantitative neuroscience. Numerous studies have used design-based stereology to enable a better understanding of the correlation of structure with function (Hédou et al., 2002; Schmitz and Hof, 2005; Zhao et al., 2016). The rapidly growing area of connectomics, previously known as neuroanatomical connections, is focused on the production of a map of connections within a specific brain region at a cellular or subcellular level (Mikula, 2016; Swanson and Lichtman, 2016). However, whether specific network arrangements can be generalized to apply across an entire brain region or between species is an area of current debate and alternative methods of assessing brain networks need to be considered (Luebke, 2017). One potential method is to use estimates of total number, of either cells or synapses within defined brain regions, from unbiased stereological estimates to establish quantitative networks at macro and micro levels (for a wider discussion of understanding of the brain as circuits see DeFelipe et al., 2016). I have suggested that the rodent brain can be considered as a distributed network with many discrete regional networks interacting to produce an immense spectrum of behavior (Koob and Volkow, 2016; Herculano-Houzel, 2017) and at each level this network is a quantitative network at a cellular and synaptic level. If all neuroscientists produced unbiased total number estimates as the key quantitative parameter this data would form the basis of a valuable data set for the construction of quantitative networks.

**Figure 1:**
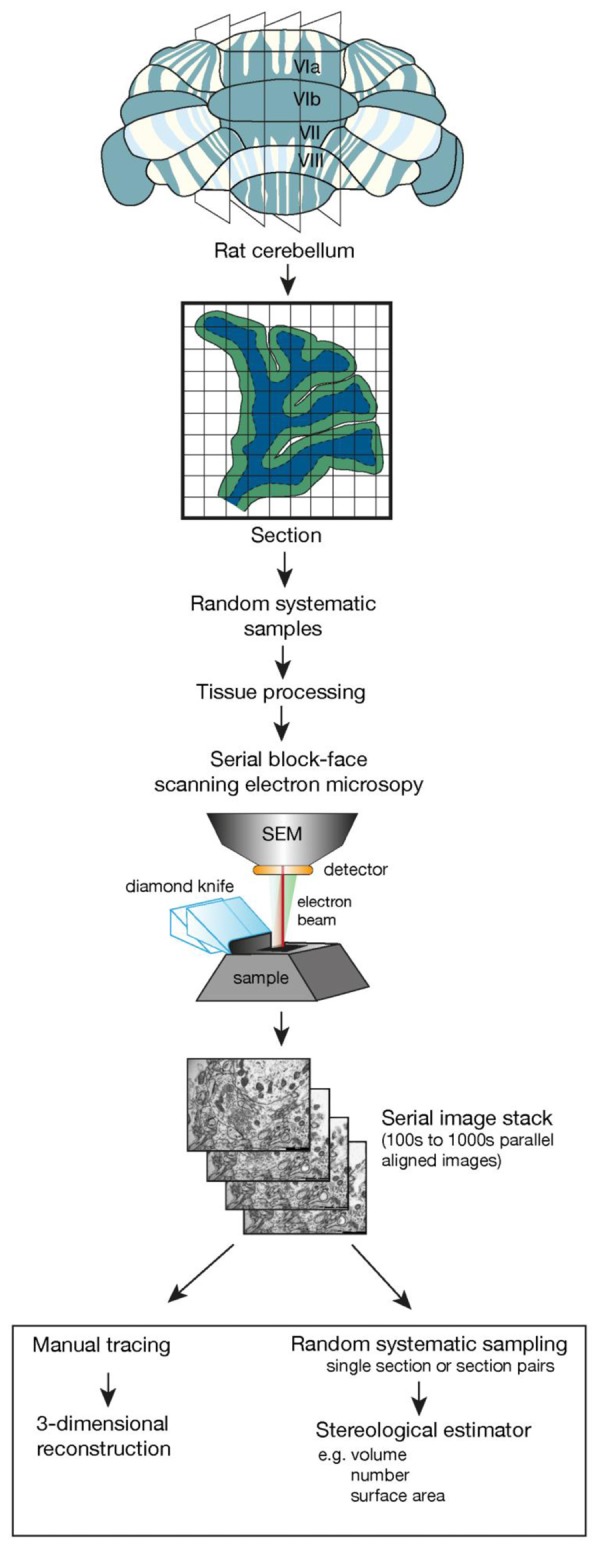
The flow diagram outlines the essential steps involved in taking a tissue sample, using random systematic sampling methodology, to the final stage of using stereological sampling probes on sections or section pairs taken from a stack of sections imaged using serial block face scanning electron microscopy. This could also be accomplished using focused ion beam scanning electron microscopy (FIB-SEM). Key elements in this design are the generation of parallel sections followed by random systematic sampling to obtain tissue samples for tissue processing and electron microscopic investigation. Random systematic sampling would then be used to obtain the sampling location within the scanning electron microscope. The cube of tissue and the number of samples imaged would be experiment dependent but each imaging location would generate a set of serial, perfectly aligned sections that could then be used with a range of stereological estimators.

Estimates of total synapse number obtained with the disector method have contributed to our understanding of network interactions in the rodent brain. This has always been technically demanding and time consuming but we are now poised with recent technical developments in electron microscopy to revolutionize the generation of quantitative synapse data. Serial block face scanning electron microscopy (SBF-SEM) and focused ion beam SEM (FIB-SEM) enable the collection of large three-dimensional data sets of nervous tissue components, at high resolution, in the electron microscope with an efficiency incomprehensible compared to conventional serial sectioning (Kim et al., 2013; Wu et al., 2017). In both SBF-SEM and FIB-SEM the high energy electrons originating in the electron beam reflected from the specimen volume (the tissue in the resin block) are captured to form the image. After each image is captured, a thin layer is removed from the block face, equivalent to or considerably less than the thickness of a conventional thin section, and the resulting new face is re-imaged. The direct imaging of the block face avoids the deformation and loss of sections that is common when images are collected from serial sections and images are sequentially built up in near perfect alignment. A set of images can be collected in a fully automated manner that previously required several years of painstaking highly skilled work (Merchán-Pérez et al., 2009; Peddie and Collinson, 2014; Wu et al., 2017). This volume of tissue generated can then be sampled using unbiased stereological methods as if it were a physical tissue block, and quantitative estimates of a range of parameters can be obtained as outlined in Figure 1 (Ferguson et al., 2017). An added value is that the three- dimensional reconstruction of components within the tissue volume enables the investigator to verify the appearance of a structure in single sections ensuring a high degree of accuracy in profile identification (see Harris et al., 2015 for further details). Validation of stereological estimates has always required comparisons with total number from reconstruction studies and this is now feasible on the ultrastructural level (Delaloye et al., 2009).

The potential use of stereological tools on SBF-SEM image stacks can be seen by considering a recent article that investigated the effects of a selective serotonin reuptake inhibitor on the dentate gyrus granule cells (Kitahara et al., 2016). It found a significant increase in extremely large spines, without an increase in spine density. The increase in spine size was accompanied by an increased postsynaptic density that correlated with an increase in volume of the presynaptic bouton and the volumes of mitochondria and synaptic vesicles within it. Potential evidence of a structural change supporting the enhanced glutamatergic neurotransmission was also detected.

Although this article used 3-dimensional reconstruction to obtain the data, stereological tools for estimating volume and surface area could be obtained in combination with the disector method for particle selection and provide data equivalent to that obtained via detailed reconstruction (Kitahara et al., 2016). It is important that we consider the use of SBF-SEM and FIB-SEM in combination with design-based stereology to advance our understanding of the ultrastructure of nervous tissue (Waworuntu et al., 2016). Ongoing developments in the capture of high resolution images and in the methods of reconstruction and optimization of images, will ensure that in the near future this is a very efficient process (Bellesi et al., 2015; Borrett and Hughes, 2016; Nguyen et al., 2016; Wernitznig et al., 2016).

## Conclusion

In order to understand the information processing in the rodent brain that underlies behavior, it is essential to understand the connectivity between neurons and synapses across a huge number of different microcircuits (Dennis and Thompson, 2013; Kelly and Castellanos, 2014; Mátyás et al., 2014). Estimates of total number, obtained using the disector method within design based stereological studies, allows the brain to be viewed as a network composed of many nodes that are each a complex of subnetworks. We are making advances in understanding the complexity of brain networks with the 3-dimensional reconstruction studies undertaken for connectomic analysis but it is also apparent that networks are location specific. If all estimates of cell and synapse number were obtained as total number from design-based stereological studies this data could be used collectively to advance our understanding of the networks of the rodent brain that underlie function and dysfunction. We must always remember the seminal work that demonstrated density as a potentially misleading quantity and be aware that the quality of the imaging technology used to obtain density does not alter the fact that it is a density and is potentially misleading. Conversations about the merits of using total number estimates obtained within design-based stereological studies vs. other methods must be held within the neuroscience community and we should strive to produce the most reliable data possible. The generation of data that can be used universally, such as total number estimates will enable greater advances in understanding the rodent and subsequently the human brain to be made.

## Author Contributions

The author confirms being the sole contributor of this work and approved it for publication.

## Conflict of Interest Statement

The author declares that the research was conducted in the absence of any commercial or financial relationships that could be construed as a potential conflict of interest.
